# Harnessing Diversity in Wheat to Enhance Grain Yield, Climate Resilience, Disease and Insect Pest Resistance and Nutrition Through Conventional and Modern Breeding Approaches

**DOI:** 10.3389/fpls.2016.00991

**Published:** 2016-07-06

**Authors:** Suchismita Mondal, Jessica E. Rutkoski, Govindan Velu, Pawan K. Singh, Leonardo A. Crespo-Herrera, Carlos Guzmán, Sridhar Bhavani, Caixia Lan, Xinyao He, Ravi P. Singh

**Affiliations:** International Maize and Wheat Improvement CenterTexcoco, Mexico

**Keywords:** wheat, genetic diversity, introgressions, disease resistance, pest resistance, cisgenesis, genomic selection, nutritional quality

## Abstract

Current trends in population growth and consumption patterns continue to increase the demand for wheat, a key cereal for global food security. Further, multiple abiotic challenges due to climate change and evolving pathogen and pests pose a major concern for increasing wheat production globally. Triticeae species comprising of primary, secondary, and tertiary gene pools represent a rich source of genetic diversity in wheat. The conventional breeding strategies of direct hybridization, backcrossing and selection have successfully introgressed a number of desirable traits associated with grain yield, adaptation to abiotic stresses, disease resistance, and bio-fortification of wheat varieties. However, it is time consuming to incorporate genes conferring tolerance/resistance to multiple stresses in a single wheat variety by conventional approaches due to limitations in screening methods and the lower probabilities of combining desirable alleles. Efforts on developing innovative breeding strategies, novel tools and utilizing genetic diversity for new genes/alleles are essential to improve productivity, reduce vulnerability to diseases and pests and enhance nutritional quality. New technologies of high-throughput phenotyping, genome sequencing and genomic selection are promising approaches to maximize progeny screening and selection to accelerate the genetic gains in breeding more productive varieties. Use of cisgenic techniques to transfer beneficial alleles and their combinations within related species also offer great promise especially to achieve durable rust resistance.

## Introduction

Wheat (*Triticum aestivum* L.), one of the key cereal crops, is grown on 222 million hectares worldwide and is a major source of calories and proteins globally (USDA, 2016). Wheat production has increased from 235 million tons in 1961 to an estimated 733 million tons in 2015 (FAOSTAT, 2014). The Green Revolution of 1960 and 1970s along with changes in policies, fertilizer use and advances in agronomy has stimulated wheat productivity over past decades (Ziska et al., 2012). A highly cited example is the global success of two semi-dwarf wheat varieties “Sonalika and Kalyan Sona” in the 1960s which helped wheat production advance from deficit to surplus in South Asia.

**FIGURE 1:**
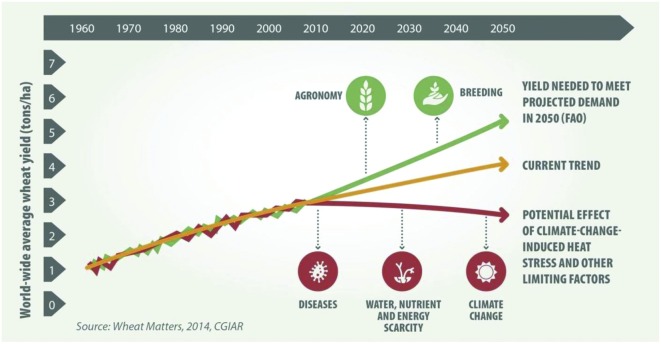
**Projected demand and yield trends in wheat under several scenarios.** Source: CIMMYT (2014).

In recent years, changes in population trends, eating habits, and economic and socio-economic conditions, especially in Africa and Asia, have caused an increase in global wheat demand. Under the assumption of favorable growing conditions, the International Grain Council [IGC] (2014) estimated the wheat production and consumption demands till 2020. Based on their predictions, wheat productivity growth was estimated at 1.1% per year for next 5 years, which will make it possible to meet the consumption demands till 2020. However, in recent years, noticeable changes in temperature and rainfall at the global level have had an impact on wheat production. Various crop models have estimated yield reductions of 6–13% in wheat for each °C rise in temperature. Based on the current trends in wheat production, the predicted increase in wheat productivity by 2050 will be short of 1 t/ha which is required to meet the rising global demand (**Figure 1**). Increased climate variability, frequent extreme weather events, and new variants of pathogens and pests further jeopardize linear productivity growth into the future. Breeding wheat for climatic change tolerance and disease resistance combined with good agronomy can potentially improve wheat productivity to meet the future demands.

Wheat is an allopolyploid species that originated from a cross of the tetraploid species *Triticum turgidum* and the diploid species *Aegilop tauschii* (Coss) Schmalh. Wild tetraploid emmer wheat evolved from a hybridization of wild *Triticum urartu* tumanian ex Gandivan and an undiscovered species of the *Aegilops speltoides* Tausch lineage. During the process of domestication genetic bottlenecks resulted in significant loss of diversity. There has been a keen interest in utilizing the genetic diversity of Triticeae species, which includes the primary, secondary, and tertiary gene pools (*Aegilops*, *Agropyron*, *Elymus*, *Hordeum*, *Leymus*, *Secale*, *Thinopyrum*, and *Triticum*). These gene pools are a rich source of genes that can be used to improve diverse traits such as disease resistance, micronutrient availability and abiotic stress adaptation. Novel alleles have been introgressed from nearly 52 species highlighting the genomic plasticity of wheat and the importance of exotic introgressions in wheat improvement (Wulff and Moscou, 2014).

In this review, we highlight the genetic diversity available in wheat for grain yield, adaptation to climate change, disease and insect pest resistance, and nutritional and end-use quality. We also discuss traditional approaches to introgression that are still successful and current technologies that are being used to characterize the genetic diversity and improve the efficiency of the introgression process. We also explore the role of new technologies such as genomic selection (GS) and cisgenesis to integrate diverse genes/alleles and accelerate the breeding process.

## Diversity in Wheat For:

### Grain Yield Improvement and Climate Resilience

Grain yield *per se* is a polygenic trait, and yield improvements from alien introgressions are due to their positive impact on phenology, yield components (that is grain size, grain number, floret number, etc.), or through adaptive traits for abiotic stresses (such as heat, drought, and alkaline/acid soils) and resistance to biotic stresses. Landraces, a crucial germplasm pool has been reported to contribute genes for grain yield improvement in irrigated environments or, in heat and drought stress environments (Reynolds et al., 2007a; Lopes et al., 2015). Direct varietal releases from simple crosses with landraces are rare, though a Turkish variety ‘Gerek 79’ is an exception (Smale and McBride, 1996). One of the best examples is the *Rht* dwarfing gene that was available through the Japanese variety ‘Norin10’ originating from a Japanese landrace Shiro Daruma (Reitz and Salmon, 1968; Dreisigacker et al., 2005). These dwarfing genes were utilized by Dr. Norman E. Borlaug to develop the high-yielding semi-dwarf wheat varieties that triggered the Green Revolution. Several other landraces also have had an impact on improving the germplasm pool: for example, ‘Cheyenne,’ a selection from landrace Crimea, founded the Nebraska wheat gene pool while ‘Turkey Red’ was used for winter wheat breeding in the USA Great Plains (Lopes et al., 2015). Studies on landraces from different regions of the world have identified potential sources for improvement of grain yield and climate resilience, for instance the drought tolerant variety ‘Aragon 03’ was developed from a selection of a landrace population ‘Catalan de Monte’ (Royo and Briceño-Félix, 2011). The potential of Mexican landraces to adapt to temperature and drought stress has been reported (Hede et al., 1999; Vikram et al., 2016). Further, allelic variation for specific plant traits such as improved 1000 kernel weight, biomass, and photosynthesis has also been identified in landraces (Lopes et al., 2015).

The development of synthetic hexaploid wheats has allowed the use of wild relatives such as tetraploid species (e.g., *Triticum dicoccum*) and the diploid species *A. tauschii* to transfer adaptive traits in to modern wheat. Genomic regions in *A. tauschii* can contribute to nearly 10% increase in grain weight (Röder et al., 2008) and improve grain yield (Börner et al., 2015). Synthetic wheats can be used to transfer such useful genetic variations. Studies have reported synthetic wheat lines that can extract more water from deeper soil, which under drought stress is an excellent adaptive trait (Reynolds et al., 2007b). Similarly, other synthetic derivatives with improved tolerance to water logging, high temperatures, and freezing have also been identified (Maes et al., 2001; Villareal et al., 2001; Yang et al., 2002).

Wild relatives of wheat also present a rich source of diversity. Species such as *Agropyron elongatum* (Host) Beauv. and *Agropyron cristatum* Gaertn. are reported to contribute to higher grain yields in wheat growing under optimal conditions. In certain wheat backgrounds, chromosome 7 Ag from *A. elongatum* increases grain yield up to 8% and carries leaf (Lr) and stem rust (Sr) resistance genes *Lr19* and *Sr25*, respectively (Singh et al., 1998). On further study this yield increase from *A. elongatum* was attributed to a better allocation of assimilates to the reproductive organs (Miralles et al., 2007). Another example is the 6P chromosome from the tetraploid species *A. cristatum*, which has been reported to increase number of florets, kernels and grain weight in wheat, in addition to improving resistance to the barley yellow-dwarf virus and powdery mildew resistance alleles (Wu et al., 2006; Wang et al., 2011).

One of the most widely used wheat relatives is rye (*Secale cereale* L.), which is well-documented as a rich source of biotic and abiotic resistance/tolerance. Rye (2n = 2x = 14), is a diploid species, originating from the Near East (Hillman, 1978; Salamini et al., 2002), belongs to the tertiary gene pool of wheat, along with *Thinopyrum* and *Elymus* species (Harlan and de Wet, 1971). The first attempts to hybridize wheat and rye were conducted by Stephen Wilson (Wilson, 1873). The first stable amphiploid triticale (*Triticosecale Wittmack*) is attributed to Rimpau in 1888; thereafter, efforts were dedicated to producing wheat-rye hybrids (Ammar et al., 2004).

Several 100s of cultivars with the (1B)1R substitution or 1BL.1RS and 1AL.1RS translocations from Petkus rye were deployed between 1960 and1990 (Rabinovich, 1998). During the 1990s, the 1BL.1RS translocation was present in 60% of wheat descending from lines developed at the International Maize and Wheat Improvement Center (CIMMYT) and nearly half of the commercial varieties (Rabinovich, 1998). In China, which is one of the major wheat growing countries, about 42% of the wheat cultivars released between 1960 and 2000 were (1B)1R genotypes, and the consistent yield gains over the years were partially attributed to the translocation (Zhou et al., 2007). Most of the desirable characteristics translocated from rye to wheat have been found in chromosome 1R that contributes to yield advantage (Villareal et al., 1998). Translocations from chromosomes 1RL and 1RS improve water use efficiency by promoting root and above ground biomass growth (Ehdaie et al., 2003; Hoffmann, 2008; Karki et al., 2014). Other rye chromosomes such as 3R, 4R, and 6R are also potential donors; introgressions from these regions could improve aluminum and acid soil tolerance in wheat.

### Disease Resistance

Diseases, caused by both fungi and fungi-like pathogens pose a major threat to wheat production. Evolution of new virulence through migration, mutation, selection, and recombination of virulence genes occurs in all pathogens, but has been more frequent in those causing rust and powdery mildew. Yield losses due to diseases can be up to 70% in susceptible varieties (Singh et al., 2008). For example, in 1998, stem rust infections were reported in Uganda caused by a new race designated as UG99. A series of reviews by Singh et al. (2006, 2008, 2011, 2015) has documented the significance, emergence, evolution and geographical spread of the Ug99 group as time progressed. Since its first discovery, 13 races within the Ug99 group have been identified across several countries in Africa and Middle East^1^. Another example in recent years is of the stripe or yellow rust pathogen. Yellow rust (Yr) is found primarily in the Northern latitudes or cooler environments, however, Hovmøller et al. (2015) found ‘Warrior’ and ‘Kranish,’ two aggressive races of yellow rust originating from sexual recombination in the near-Himalayan region of Asia which can infect host under warmer temperatures.

One of the strategies to mitigate the threat from diseases is to identify and utilize diverse sources of durable resistance. Globally important fungal diseases of wheat caused by biotrophs (obligate parasites), include the three rusts; leaf or brown rust, stripe or yellow rust and stem or black rust, caused by *Puccinia triticina*, *Puccinia striiformis* f. sp. *Tritici*, and *Puccinia graminis* f. sp. *Tritici*, respectively, powdery mildew caused by *Blumeria graminis* f. sp. *tritici*; whereas, those caused by hemibiotrophs (facultative parasites) include Fusarium head blight, *Septoria tritici* blotch, leaf blotch, spot blotch, and tan spot.

Resistance genes can be characterized as race specific and race non-specific, this classification dates back to 1962 when Van der Plank proposed the first theoretical concepts of disease resistance. Race specific genes confer resistance to one or a few races of a pathogen and are known to be based on ‘gene for gene’ interaction. Also known as ‘major genes,’ they usually have large phenotypic effects, but may not confer complete resistance. Although incorporation of race-specific resistance genes may be promising, it increases the risk of faster breakdown. Some examples of major genes for rust resistance include *Lr19*, *Lr26*, and *Lr42* effective against leaf rust, *Yr5*, *Yr10*, and *Yr15* against yellow rust and *Sr22*, *Sr26*, and *Sr35* against stem rust. Race non- specific resistance, is usually effective in the post-seedling growth stage, thus commonly referred to as adult plant resistance (APR). Race-non specific resistance is generally quantitatively inherited and ranges from moderate resistance/moderate susceptibility to nearly complete resistance and interact additively with other non-specific resistance genes. Varieties with high levels of durable resistance to multiple pathogens can be developed by combining multiple race non-specific resistance loci, especially to those which are known to confer resistance to multiple diseases (Singh et al., 2008). Examples of these pleiotropic resistance genes are *Lr34*, *Lr46*, and *Lr67* which provide resistance to leaf, yellow and stem rust and powdery mildew. Because race non-specific resistance can provide broader and robust resistance to fight pathogen evolution it has been recommended for the high risk areas, for instance in East African highlands where wheat cultivation and pathogen evolution is continuous (Singh et al., 2008).

**FIGURE 2:**
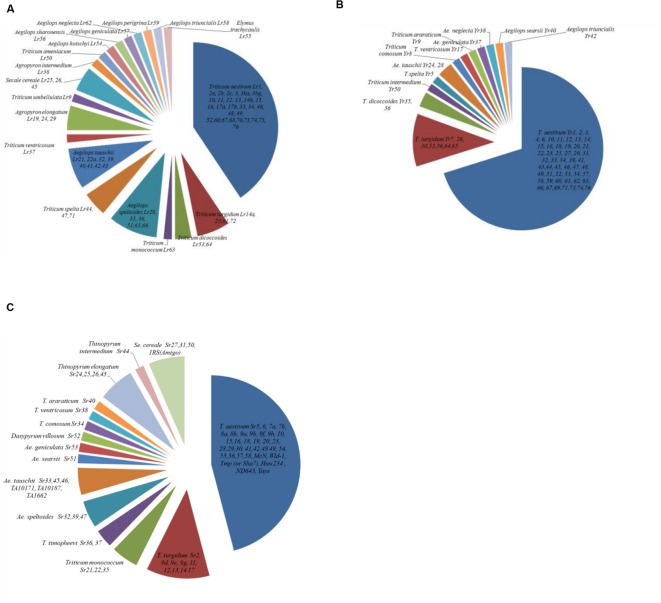
**Origin of designated **(A)** leaf rust, **(B)** stripe rust, and **(C)** stem rust genes conferring seedling and/or adult plant resistance**.

Though most rust resistance genes originated from hexaploid wheat, there are also many genes that originated from the wild relatives and other genera such as *Aegilops*, *Dasypyrum*, *Thinopyrum*, and *Secale* (**Figure 2**). As early as 1920 and 1930s, introgression of stem rust resistance from *T. turgidum* subsp. durum and *T. dicoccum* subsp. Dicoccum Schrank ex Schubler into bread wheat was reported (Hayes et al., 1920; McFadden, 1930). Both race-specific and non-specific genes have been identified from diverse genetic sources. For instance, *Lr9* from *Aegilops umbellulata* Zhuk, *Yr5* from *Triticum spelta* L., *Yr28* from *A. tauschii*, *Sr9e* from tetraploids and *Sr35* from *Triticum monococcum* L. are race-specific genes. Examples of race non-specific genes/APR include *Lr22a* from *A. tauschii*, *Yr36* from *Triticum diccocoides* (Korn. Ex Asch. and Graebn) Schweinf, *Yr48* from synthetic hexaploid wheat PI610750 and *Yr52, 56, 57*, and *62* from landraces. Introgressions are also associated with multiple disease resistance as well, such as *Pm8/Sr31/Lr26/Yr9* from rye, *Sr36/Pm6* from *Triticum timopheevi* (Zhuk.) Zhuk., Pch1 and *Sr38/Lr37/Lr17* from *Aegilops ventricosa* Tausch, and *Lr19/Sr25*, *Sr24/Lr24*, and *Sr26* from *A. elongatum* (Host) P. Beauy (Sears, 1956; Friebe et al., 1996; Mago et al., 2005; Wulff and Moscou, 2014). Some genes introgressed from wild relatives have been associated with negative linkage drag and therefore have not been widely deployed in breeding: examples include *Sr32* and *Sr37* identified in *A. speltoides* (McIntosh et al., 1995) and *T. timopheevi* (McIntosh and Gyarfas, 1971) respectively. Other temporarily designated genes that are common in high yielding wheat germplasm offer additional possibilities for combining resistance genes combinations.

Novel alleles from genetically diverse sources have also been identified for other important wheat diseases. For example, Fusarium head blight resistance genes are from genera *Roegneria*, *Hystrix*, *Elymus*, *Kengyilia*, and *Agropyron* (Wan et al., 1997) and other related species, e.g., *T. timopheevi*, *T. monococcum*, *Triticum karamyschevii* Neyski, and *T. militinae* Zhuk and Migush (Cai et al., 2005). Genes conferring powdery mildew resistance have been reported from *T. dicoccoides* (Moseman et al., 1984), *Triticum carthlicum* Nevski, *T. monococcum* and *T. timopheevi* (Tomerlin et al., 1984). Some of the designated genes for resistance to powdery mildew, fusarium head blight and *Septoria tritici* blotch are given in **Table 1.** Wheat blast caused by *Magnaporthe oryzae* (anamorph. *Pyricularia oryzae*) is an emerging disease in the tropical parts of the Southern Cone of South Americas and was reported in Bangladesh as well. Though wheat blast is a recent disease, resistance has been identified in *A. tauschii* (Bockus et al., 2012) and in synthetic wheats (Cruz et al., 2010). The 2NS/2AS translocation from *A. ventricosa* was recently found to confer wheat blast resistance (Cruz et al., 2016), though unpublished reports from Paraguay have documented the emergence of new isolates virulent to this resistance. Both qualitative and quantitative resistance have been observed and the former has been validated at the seedling stage (Maciel et al., 2014). So far, eight resistance genes have been identified (i.e., *Rmg1* to *Rmg8*), of which only *Rmg2*, *Rmg3*, *Rmg7*, and *Rmg8* are host resistance genes against *Triticum* isolates of *Pyricularia oryzae*; the rest are non-host resistance genes (Anh et al., 2015). It is noteworthy that only *Rmg7* was identified in *T. dicoccum* (Tagle et al., 2015) whereas all are from bread wheat (Anh et al., 2015). Thus diverse resistant sources are available for both the existing and the emerging diseases in wheat.

**Table 1:** Known genes for resistance to Powdery mildew, Fusarium head blight and *Septoria tritici* blotch from landraces, wild relatives and synthetic wheat.

Diseases	Source of Resistance	Genes
Powdery mildew (*Blumeria graminis* f. sp. *tritici*)	*Triticum monococcum*	*Pm4d*, *Pm1b*, and *Pm1c*
	*Triticum urartu*	*PmU*
	*Triticum boeoticum*	*Pm25*, *PmTb7A.1*, and *PmTb7A.2*
	*Triticum dicoccoides*	*Pm16*, *Pm26*, *Pm30*, *Pm31*, *Pm36*, *Pm41*, *Pm42*, and *MeIW72*
	*Triticum dicoccum*	*Pm4a*, *Pm5a*, *Pm49*, and *Pm50*
	*Triticum carthlicum*	*Pm4b*, *Pm33*, and *Pm46*
	*Triticum spelta*	*Pm1d*, *Pm10*, and *Pm11*
	*Triticum sphaerococum*	*Pm3b* and *Pm36*
	*Triticum timopheevi*	*Pm6*, *Pm27*, and *Pm37*
	*Aegilops tauschii*	*Pm2*, *Pm19*, *Pm34*, and *Pm35*
	*Aegilops speltoides*	*Pm12* and *Pm32*
	*Aegilops longissimi*	*Pm13*
	*Aegilops ovata*	*Pm29*
	*Secale cereale*	*Pm7*, *Pm8*, *Pm17*, and *Pm20*
	*Thinopyrum intermedium*	*Pm40* and *Pm43*
	*Haynaldia villosum*	*Pm21*
Fusarium head blight (*Fusarium graminearum*)	*Triticum* spp.	*Fhb1, Fhb2, Fhb4*, and *Fhb5*
	*Leymus rasomsus*	*Fhb3*
	*Elymus tsukushiensis*	*Fhb6*
	*Thinopyrum ponticum*	*Fhb7*
*Septoria tritici* blotch (*Mycosphaerellla graminicola*)	Synthetic Wheat (Synthetic 6x, W7984, M3)	*Stb5, Stb8, Stb16q*
	*Triticum monococcum* (W7984)	*TmStb1*

*Friebe et al. (1996) and McIntosh et al. (2013).*

### Insect Pest Resistance

It is estimated that global yield losses due to insect pests in the pre-green revolution era were about 5.1%, however, the losses increased to 9.3% in the post-green revolution in 1990s (Dhaliwal et al., 2010). Insect pests are dynamic and highly adaptable. Changes in environmental temperature can modify their physiology, behavior, voltinism, and distribution. For instance, with warmer winters, the number of aphid generations per wheat growing cycle may increase (Hullé et al., 2010) and extend their distribution further (Macfadyen and Kriticos, 2012). It has also been proven that aphids can modify their behavior in response to either high or low temperature stress (Ma and Ma, 2012; Alford et al., 2014), enabling them to adapt in the presence of natural selection if genetic variation exists for such traits. While the work on disease resistance has tremendously contributed to protect wheat yields, control of arthropod pests has largely depended on the use of chemicals. A dramatic positive impact could be achieved through the introduction of new resistance genes (either singly or in combination with of multiple genes) to provide a broad spectrum of protection against multiple pathogens and insect biotypes.

There are several examples where genes from alien sources have been found to confer resistance to some of the most important wheat pests such as aphids *Schizaphis graminum* (Rondani), *Diuraphis noxia* (Mordvilko), *Rhopalosiphum padi* L. and *Sitobion avenae* (F.), the cecidomyid *Mayetiola destructor* (Say), the nematode *Heterodera avenae* (Wollenweber) and the mite *Aceria tosichell* Keifer. Several wheat-related species have been found to be resistant to aphids; however, efforts to incorporate such resistance sources into wheat breeding pipelines are limited and there are only few a specific cases in which aphid resistant cultivars are purposely bred (i.e., *D. noxia* in the USA and South Africa, and *S. graminum* in the USA). To determine the utility of such genetic resources for aphid resistance, Smith et al. (2004) evaluated 21 accessions from six species of *Aegilops* and one accession of *Triticum araraticum* Jakubz that were previously identified to be resistant to *R. padi* and found antibiotic effects on *S. avenae* and *D. noxia* in an *Aegilops neglecta* accession. Migui and Lamb (2003) evaluated resistance to *R. padi*, *S. avenae*, and *S. graminum* in 19 species related to wheat, and found that species such as *Triticum boeoticum* Boiss., *A. tauschii* and *T. araraticum* had the higher levels of resistance to *R. padi*, whereas *A. tauschii* and *T. turgidum* had higher levels of overall resistance to *S. graminum*, and *T. araraticum* and *T. dicoccoides* had higher levels of overall resistance to *S. avenae*. However, for other destructive pests such as, *Eurygaster integriceps* Puton, more work is required to find adequate resistance levels that can be incorporated in wheat cultivars (El Bouhssini et al., 2009). Friebe et al. (1996) made a comprehensive review of wheat-alien translocations that confer resistance to wheat biotic stresses. Here, some examples of resistance to diseases and pests translocated from rye are reviewed (see **Table 2**, where we summarize resistance sources by rye chromosome and diseases/pests).

**Table 2:** Examples of resistance genes for diseases and pests from rye (*Secale cereale*).

Diseases	Gene	Description	Germplasm
Leaf rust (*Puccinia triticina*)	*Lr26*	1BL.1RS	Petkus rye; Kavkaz and Veery wheat derived
	*Lr25*	4BS.4BL-2RL	Transec
	*Lr45*	2AS-2RS.2RL	RL6144
Stripe Rust (*Puccinia striiformis* var. *striiformis*)	*Yr9*	1BL.1RS	Petkus rye; Kavkaz and Veery wheat derives
	*YrCN17*^†^	1BL.1RS	R14, Chuan-nong 17
	*YrR212*^†^	1BL.1RS	R212
Stem rust (*Puccinia graminis* f. sp. *tritici*)	*Sr31*	1BL.1RS	Petkus rye; Kavkaz and Veery wheat derives
	*Sr50/SrR*	1BL.1RS	Imperial rye derives
	*Sr1RS^Amigo^*	1AL.1RS	Amigo wheat
	*Sr27*	3AL.3RS	WRT238
Powdery mildew (*B. graminis* f. sp. *tritici*)	*Pm8*	1BL.1RS	Petkus rye; Kavkaz and Veery wheat derives
	*Pm17; allelic to Pm8*	1AL.1RS	Insave rye derives; Amigo wheat derives
	*Pm7*	4BS.4BL-2RL	Transec
	*Pm20*	6BS.6RL	WGRC28
Greenbug (*Schizaphis graminum*)	*Gb2*	1AL.1RS	Insave rye, Amigo wheat derives
	*Gb6*	1AL.1RS	Insave rye, GRS1201
*Diuraphis noxia*	*Dn7*	1BL.1RS	94M370 wheat
Hessian fly (*Mayetiola destructor*)	*H21*	2BS.2RL	KS85HF 011-5
	*H25*	4BS.4BL-6RL	Balbo rye; 88HF16 wheat
*Aceria tosichell*	*CmC3*	1AL.1RS	Amigo wheat
Cereal cyst nematode (*Heterodera avenae*)	*CreR*	6DS.6RL	T-701 triticale derives

*^†^Temporary designation.*

*Friebe et al. (1996) and McIntosh et al. (2013).*

### End-Use Quality and Nutritional Quality

In addition to combating abiotic and biotic stresses while improving grain yield, wheat breeding must improve or at least maintain the nutritional and end-use quality. The wide variety of food products made from wheat flour has resulted in ongoing demand from the wheat processing industry for wheat with specific quality attributes. Additionally, dietary deficiencies of essential micronutrients such as zinc (Zn) and iron (Fe) are a major health concern in developing countries especially for pregnant women and children under age 5. An estimated 17.3% of the world’s population is at risk for inadequate zinc intake, a factor highly correlated with stunted growth in children (Wessells and Brown, 2012). Genetic biofortification with natural genetic variation present in wild relatives, synthetics and landraces for micronutrient uptake from the soil and translocation in to wheat grain is a sustainable solution that can supplement micronutrient-deficient rural inhabitants with limited access to formal markets or health care systems (Velu et al., 2014).

**FIGURE 3:**
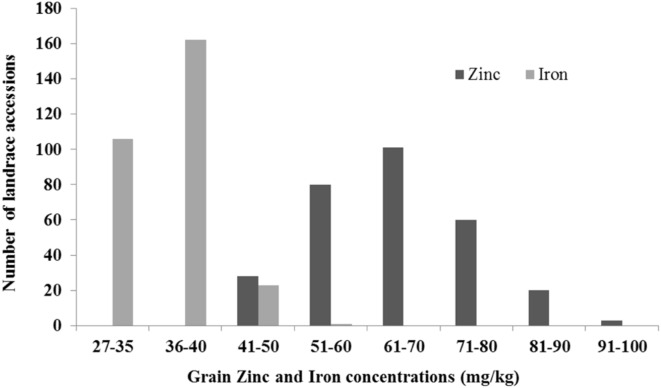
**Genetic diversity for grain Zinc and Iron in Mexican and Iranian landrace collections**.

In recent years, the focus has been on “biofortification” of wheat with micronutrients, specifically Zn and Fe. Evaluation of landraces and secondary gene pools (i.e., tetraploid and diploid progenitors of hexaploid wheat) for micronutrient concentration identified *T. dicoccoides, A. tauschii, T. monococcum*, and *T. boeticum* Boiss. as the most promising sources for improving Fe and Zn grain concentration (Cakmak et al., 2000; Monasterio and Graham, 2000). Large scale screening of available wheat genetic resources at CIMMYT identified einkorn wheat, wild emmer wheat, and landraces with high amounts of Zn and Fe in grain (Cakmak et al., 2000; Ortiz-Monasterio et al., 2007). The available genetic variation in wild emmer (*T. dicoccoides*), *T. spelta, T. dicoccum* species is being used to develop nutrient-enriched wheat germplasm. The stocks (*T. turgidum* ssp. *dicoccum*/*A. tauschii*) are also being used for genetic biofortification of Zn and Fe by CIMMYT’s wheat breeding program (Ortiz-Monasterio et al., 2007; Morgounov et al., 2007). Recently, evaluation of a representative subset of Mexican and Iranian landraces under Zn-enriched soil conditions in Cd. Obregon, Mexico, showed more than a twofold variation for Zn (40–96 mg/kg) and Fe (27–56 mg/kg; **Figure 3**). A major locus affecting Zn and Fe concentration, *Gpc-B1* (250 kb-locu), was mapped, and found to encode a NAC transcription factor (NAM-B1) that accelerates senescence and increases nutrient remobilization from leaves to grain (Uauy et al., 2006; Distelfeld et al., 2007). Interestingly, the favorable allele of *Gpc-B1* is from *T. dicoccoides* and all modern tetraploid and hexaploid wheats possess a non-functional allele of NAM-B1, indicating that the NAM-B1 function was lost during domestication.

Apart from micronutrients, wheat grain is also a good source of other beneficial nutrients which could be targeted by breeding programs to improve the nutritional quality of wheat based products. Grain bran is particularly rich in dietary fiber, vitamins (folic acid), and phytochemicals, which have been associated with a protective role for many chronic diseases including cardiovascular diseases and type 2 diabetes (Jacobs et al., 1999; Liu et al., 1999; de Munter et al., 2007). The HEALTHGRAIN cereal diversity screening project reported diversity for dietary fiber and phytochemicals in the wheat primary gene pool. The levels of dietary fiber ranged from 11.5 to 18.3% of dry matter, and more specifically the content of water extractable arabinoxylans (an important source of soluble dietary fiber, which is more readily fermentable in the colon than insoluble one) ranged from 0.3 to 0.85% in bran and from 0.3 to 1.4% in flour (Gebruers et al., 2008; Ward et al., 2008; Kariluto et al., 2010). Various research projects are currently ongoing to screen for genetic variability of the bioactive compounds (Di Silvestro et al., 2012; Giambanelli et al., 2013; Laddomada et al., 2016). High heritability for some of these compounds such as tocols, sterols and arabionoxylan fiber (Shewry et al., 2010) and available genetic diversity increases the chances of utilizing the variation for improving nutritional quality in wheat.

The vast catalog of products prepared from wheat requires genetic variation in traits related to grain composition as well. Exploring novel genetic variation could improve processing and end-use quality. Grain proteins are one of the important components that influence end-use quality. Studies have reported higher grain protein content in landraces than in modern wheat (Rodriguez-Quijano et al., 1994; Dotlacil et al., 2010) which means landraces and wild relatives could be a potential source to improve protein content. In fact, as mentioned above *GPC-B1* (also called *NAM-B1*), the first gene identified for grain protein content variation was transferred from a wild emmer accession (*T. dicoccoides*) to modern durum wheat background (Avivi, 1978; Joppa and Cantrell, 1990; Joppa et al., 1997). While grain protein content is important, gluten quality is equally important. Gluten, an essential component of dough, is a complex protein network formed mainly by two kinds of proteins, monomeric gliadins and polymeric glutenins, which in turn are divided into high molecular weight glutenins (HMWGs) and low molecular weight glutenins (LMWGs). Although there is allelic variation in modern wheat for the gene *Glu-1* encoding HMWGs, use of diversity in the Triticeae pool could potentially contribute to improve processing quality (Xu et al., 2010; Rasheed et al., 2014). The Wheat Gene Catalog currently describes 26 alleles for *Glu-A1*, 56 for *Glu-B1*, 24 for *Glu-D1*, 55 for *Glu-A3*, 32 for *Glu-B3*, and 16 for *Glu-D3* (McIntosh et al., 2013) Several of those alleles have been detected in modern wheat ancestors and wild relatives, such as *Glu-B1q* in emmer (Vallega and Waines, 1987), *Glu-B1be* in wild emmer (Xu et al., 2004), *Glu-D1n* in spelt (Caballero et al., 2001), or *Glu-D1bf* in *A. Tuaschii* (Gianibelli et al., 2001). Genetic resources (for example *T. urartu* or *T. monococcum*) can be utilized to introgress *Glu-A1 x+y or y* active subunits (always silenced in modern durum and bread wheat, respectively), which will lead to new variations (Alvarez et al., 2009). Recently, a novel allele HMW glutenin allele was identified from *A. longissimma* Schweir and Muschl through the use of a Chinese Spring substitution line CS-1S(1B) that could potentially improve dough and breadmaking quality (Wang et al., 2013).

Other important quality traits such as grain hardness or starch properties are also influenced by diverse proteins and therefore genes. Puroindolines a and b (PINA, PINB), encoded by the genes *Pina-D1* and *Pinb-D1*, are responsible for grain hardness (Morris, 2002). Wild alleles of *Pina-D1a* and *Pinb-D1a* are linked to soft grain texture, though several alleles for both *Pin-D1* genes have been associated with harder grain in modern wheat (Giroux and Morris, 1997, 1998; Lillemo and Morris, 2000; Ikeda et al., 2010), landraces (Chen et al., 2005, 2007; Ayala et al., 2013) and wild relatives (Massa et al., 2004; Guzmán et al., 2012; Cuesta et al., 2013). It is interesting to note that some of these alleles have been associated with differences in quality traits other than grain hardness (Brites et al., 2008; Tanaka et al., 2008; Chen et al., 2013). While knowledge on the diverse sources for genes to improve end-use quality is available, utilization of the diversity within the breeding programs is not prevalent.

## Harnessing Diversity in Wheat

### Traditional Breeding Approaches

The success of breeding to introgress beneficial genomic regions into wheat is conditioned by the relatedness between the species (Friebe et al., 1996). Mujeeb-Kazi and Wang (1995) identified certain key requirements for introgression, (1) the genome constitution of the donor species; (2) the genomic relationship between the donor and recipient species; (3) chromosomal location of the loci of interest; (4) whether the gene(s) of interest can be expressed in the recipient species; and (5) whether gene transfer has any negative effect on the recipient species. For instance, introgression can be achieved by direct hybridization, homologous recombination, backcrossing and selection if the donor species belongs to the primary gene pool, e.g., hexaploid landraces, cultivated tetraploids (*T. turgidum*), wild emmer wheats (*T. dicoccoides*) or diploids *T. monococcum* and *A. tauschii*. If the donor species belongs to the secondary gene pool (e.g., polyploid *Aegilops* and *Triticum* species, and the S-genome species of the genus *Aegilops*) homologous recombination is possible if the loci of interest are transferred in homologous chromosomes. For species belonging to the tertiary gene pool (e.g., *Elymus* species), gene transfer can be achieved by exploiting the centric breakage-fusion of univalents, induced homoeology and radiation treatment to induce chromosome breaks (Friebe et al., 1996; Feuillet et al., 2008). Synthetic hexaploid wheats carry novel variation for tolerance/resistance to abiotic and biotic stresses but are usually poor in agronomic performance. While they are used for transferring useful genetic variation into common wheat, typically one or two backcrosses to elite germplasm followed by selection are required to identify lines with superior performance.

Although such introgressions can be of benefit to wheat, the donor sources often negatively impact previously selected adaptation traits in the recipient germplasm because alien chromatin is usually incorporated as large blocks that may carry alleles associated with undesirable agronomic characteristics. Depending on the wheat genetic background, the rye source and the type of abiotic stress factors, studies have shown that rye transferred into wheat may have both positive and negative effects on wheat performance. Monneveux et al. (2003) reported that depending on the wheat background, 1BL.1RS translocations can negatively impact yield under rainfed conditions and heat stress. However, in general, under non-stressed conditions, 1RS confers higher yield regardless of which wheat chromosome (1A, 1B, or 1D) it is translocated into (Kim et al., 2004). On the other hand, the position of 1RS in the wheat genome can negatively affect baking quality, thus genotypes with 1AL.1RS and 1DL.1RS are preferred over genotypes with 1BL.1RS (Graybosch et al., 1993; Kim et al., 2005). Traditional breeding methods such as repeated backcrossing and selection of desirable genotypes often require extensive efforts and are time consuming. However, with the new advances in phenotyping, QTL mapping, and genetic modification, along with sequencing technologies are expected to improve the precision and speed of alien introgression (Jacobsen and Schouten, 2007; Tiwari et al., 2014).

## Modern Breeding Approaches

### High Throughput Phenotyping

Phenotypic characterization is important prior to the efficient utilization of genetic diversity. Most phenotypic traits, heading time, photoperiodic responses and vernalization responses are explained by the germplasm’s geographical origin (Kato and Yokoyama, 1991; Cavanagh et al., 2013). Phenotyping for agronomic traits, response to disease and pests and other adaptive traits is crucial for the introduction of new allelic variation in breeding programs. Targeted characterization of germplasm panels such as the Focused Identification of Germplasm Strategy (FIGS), developed based on agro-ecological data enables identification of specific adaptive traits within the genetic resources. For instance, Reynolds et al. (2015) applied FIGS set to evaluate landraces, and found that those from heat and drought stressed regions had 40% greater biomass under heat and drought compared to modern varieties.

Greenhouse based automated phenotyping platforms using robotics and sensor imaging are being used for data acquisition in different crops by a number of institutes globally (e.g., IPK Gatersleben, Germany and The Plant Accelerator, Adelaide, Australia). Though, the high operational cost of such high throughput phenotyping platforms limits their large-scale use in breeding programs. Recent developments in remote sensing and high throughput phenotyping technologies allow characterization of a large number of germplasm in a short amount of time. Spectral imagery can be utilized to measure normalized difference vegetation index (NDVI), canopy temperature, hydration status, and pigment composition (Honsdorf et al., 2014; Rahaman et al., 2015; Reynolds et al., 2015). These spectral indices have already been linked to ground based measurements of yield, biomass, and adaptation (Reynolds et al., 1994). Availability of high-resolution cameras has made it possible to focus on phenotypic characterization at the plot level. For instance, spectral indices estimated by using low level airborne remote sensing showed significant association with those collected at ground level (Tattaris et al., 2013). Along with advances in statistical modeling methods, it is possible to predict plant performances in the field, based on the information obtained from high-throughput phenotyping. Such technologies could be used for characterization of the diverse germplasm pools to identify potential sources for tolerance/resistance to abiotic and biotic stresses.

### Genome Wide Association Mapping and Marker Assisted Backcrossing

The use of molecular markers for identifying functional genes and genome wide association studies (GWAS) can greatly facilitate the introgression process. GWAS studies on landraces and wild relatives of wheat have identified quantitative trait loci (QTL) associated with morphological traits in normal irrigated, heat and drought environments and with disease resistance (Kertho et al., 2015; Liu et al., 2015; Sukumaran et al., 2015). If large effect QTL exist for traits of interest and the favorable alleles originate from exotic sources, then marker assisted backcrossing (MABC) can be used to more rapidly introgress such alleles into elite backgrounds compared to conventional backcrossing (Hillel et al., 1990; Tanksley and Nelson, 1996).

Marker assisted backcrossing involves selecting of favorable alleles using QTL linked markers during each backcrossing generation. To reduce the number of backcrossing generations required to recover the recurrent parent genome, markers distributed across the genome can be used to select individuals with the favorable donor QTL and the highest proportion of recurrent parent genome (Young and Tanksley, 1989; Hillel et al., 1990, Hospital et al., 1992). This approach, referred to as MABC with foreground and background selection, can be highly effective with availability of gene based markers and markers tightly linked to QTL determine (Ellis et al., 2014). This approach has been suggested for improving a wide range of traits conferred by large effect genes, including rust resistance genes in wheat. If QTL positions are uncertain (such is the case of positions inferred by QTL mapping studies), then flanking markers located several centimorgans on either side of the QTL are needed to ensure the QTL is not lost during backcrossing (Visscher et al., 1996). This may be problematic if there is linkage drag associated with the QTL, and large flanking segment may inevitably be introgressed. Fine-mapping or cloning the QTL to develop closely linked or functional markers would be ideal for backcross introgression from exotic germplasm. Unfortunately, in wheat, fine mapping and cloning can take several years.

In addition to certainty of QTL positions and availability of tightly linked markers, the number of targeted QTL is another factor that should be considered before attempting MABC. The proportion of single MABC progeny containing donor alleles at all QTL is 0.5n, where n is the number of QTL and assuming QTL are unlinked, and the position of the QTL is known with certainty. For example, to introgress of 5 QTL, approximately 3% of the progeny can be expected to contain all favorable alleles; thus 145 progeny would be required obtain one individual with all three alleles with a 1% risk of failure. Reducing linkage drag when introgressing multiple QTL can be hastened dramatically when using background selection to identify the desired recombinants. However, the probability of observing the desired recombinants remains low, and several generations of backcrossing may ultimately be needed. To introgress multiple QTL, a QTL pyramiding scheme where QTL are first introgressed in the desired background singly and then combined would be more efficient (Hosptial and Charcosset, 1997). An algorithm for designing optimal gene or QTL pyramiding schemes was presented by Servin et al. (2004).

Marker assisted backcrossing is being applied at CIMMYT to improve grain Zn and Fe concentrations. Various studies have reported QTL for high grain Fe and Zn concentrations on chromosomes 1A, 2A, 2B, 3D, 4B, 6A, 6B, and 7A in different species of diploid, tetraploid, and hexaploid wheat (Peleg et al., 2009; Tiwari et al., 2009; Xu et al., 2012; Hao et al., 2014; Srinivasa et al., 2014). A recombinant inbred line (RIL) population developed from the cross between ‘PBW343’ and ‘Kenya Swara’ was used to identify QTL and markers associated with Zn. Two novel large effect QTL on chromosomes 2B and 3A were successfully converted into usable form for marker assisted introgression of this QTL in to an elite background. During the 2014–2015 crop season, selected RILs that showed significantly enhanced Zn compared to either of the parental lines PBW 343 or Kenya Swara was used to transfer the QTL of interest using foreground selection. This strategy will serve to move desirable alleles rapidly and precisely into the adapted background.

### Genomic Selection

When the number of QTL is large, MABC and pyramiding schemes may not be feasible. Phenotypic selection is currently the most reliable and widely used method for introgressing of favorable alleles from an exotic, non-adapted parent. GS techniques can also be applied to increase the rate of genetic gain in populations derived from exotic and elite parents. As reviewed by Lorenz et al. (2011), GS is a marker assisted breeding method in which genome wide markers and phenotypes from a reference population are used to train a prediction model. That prediction model is then used to predict breeding values based only on their genome-wide marker data. GS is more effective than MAS or marker assisted recurrent selection for polygenic traits (Bernardo and Yu, 2007). To achieve good prediction accuracy, it is important that the model training population be representative of the selection candidates that are to be predicted (Hayes et al., 2009; Pszczola et al., 2012).

If exotic parents are used in the breeding program, then an existing model training population will not be effective for predicting the progeny of these crosses. If one is to use GS to select among progeny from an exotic by elite cross, then a subset of the progeny will need to be phenotyped for model training. That prediction model could then be used for a few generations of recurrent selection within the bi-parental population. If the objective is to backcross favorable alleles from the exotic parent into an elite background, then GS can be used to identify the backcross progeny to cross to the recurrent parent. A simulation study by Bernardo (2016) found that the most effective GS backcrossing approach to introgress QTL from an exotic into an elite background was to train the GS model using F2 progeny and then apply that model during multiple generations of backcrossing. This approach led to a greater selection compared to phenotypic selection or selection based on QTL linked markers alone.

If crossing with exotic parents without backcrossing or within family recurrent selection, then it would be best to refrain from GS among families where one of the parents is exotic or exotic-derived until a sufficient number of progeny and other relatives descending from the exotic have been phenotyped. GS for allele introgressions has not yet been attempted in wheat; however, the use of GS in breeding with elite germplasm has shown significant potential. There are at least 29 studies on GS in wheat that have been published. Two studies (Heffner et al., 2011a,b) showed the potential of this approach to predict end-use quality traits of soft bread wheat germplasm, and obtained promising results, although forward prediction of quality traits was not carried out. A 5-years study conducted at CIMMYT with elite breeding lines for flour quality reported forward prediction accuracies of 0.68 and 0.49 for aleveograph W and loaf volume respectively (Battenfield et al., 2016). In another GS study a cross-validation of genomic predictions revealed moderately high predictability for grain Zn(0.5) and Fe(0.6) (Velu et al., 2016). Several cross validation studies have assessed the potential to use GS for improving disease resistance (Ornella et al., 2012; Rutkoski et al., 2012, 2015; Daetwyler et al., 2014; Arruda et al., 2015; Mirdita et al., 2015) and for grain yield (Crossa et al., 2010; Poland et al., 2012; Dawson et al., 2013) in wheat.

### Next Generation Approaches

The concept of cisgenesis was defined by Schouten et al. (2006) as the transfer of genes within the gene pool of sexually compatible species of same genus. Though similar to classical breeding, this approach has the potential to overcome its two major limitations. Cisgenesis can be used to hasten the transfer of targeted genes between related species and can avoid linkage drag associated with classical breeding. The strategy can also be used to improve traits with limited natural allelic variation in the gene pool. Higher expression of the traits can be obtained by re-introducing the gene with its own promoter and terminator or expression levels can be lowered through silencing constructs (Holme et al., 2013). In the case of wheat, cisgenic transfers are limited within the *Triticum* genus, though the availability of triticale, a hybrid between rye and wheat, and hybrids between barley and wheat, opens up new opportunities for cisgenesis between two divergent sexually compatible gene pools, *Triticum–Secale* and *Triticum–Hordeum* (Holme et al., 2013). There are a few of examples of the use of cisgensis in cereals. The HMW glutenin subunit 1Dy10 associated with superior bread-making quality is present in hexaploid bread wheat but absent in durum wheat. Cisgenesis was used to transfer the *1Dy10* HMW glutenin gene from bread wheat to durum wheat (Gadaleta et al., 2008). Further work is ongoing to improve phytase activity in barley (Kerr et al., 2010) and drought tolerance in ryegrass (Bajaj et al., 2008).

With evolution of technologies, introgression of multiple genes as cassettes through cisgenesis is also being explored. Extensive time and effort is required to transfer multiple genes from genetically diverse sources in to cultivated varieties, requiring multiple backcrosses and selection against undesirable traits. The development of gene cassettes could potentially solve the issues related to sexual incompatibility, linkage drag and introgression from other genera. Wulff and Moscou (2014) described it to be equivalent to the wheat-rye translocation (1BL:1RS) which harbors different genes for disease resistance. Ellis et al. (2014) suggested constructing gene cassettes with multiple resistance genes combined resistance against the three rust diseases will result in durable resistance in wheat. The ability to produce cassettes will allow combining genes that cannot be selected in a normal breeding processes or introgress genes linked in repulsion; this will lead to rapid introgression into cultivars. Though the new technologies show great potential, they have several limitations. Both the cisgenesis and gene cassettes approaches will require genome-editing technologies that are still under development. Furthermore, there may be issues with gene suppression or loss of gene expression due to host gene interaction. For example, Hurni et al. (2014) observed that the powdery mildew gene *Pm3* in wheat suppresses its ortholog *Pm8* transferred to wheat from diploid rye due to interactions of encoded proteins, thus limiting transfer of multiple genes for resistance. Finally, government regulations and acceptance within the scientific and social community will drive the application of these technologies in wheat breeding.

## Conclusion

The rich genetic diversity available in wheat is a source of numerous novel alleles for grain yield, disease resistance and tolerance to abiotic stress. While scientists realized the importance of genetic diversity decades ago, there is still a huge gap in characterization of the available genetic resources and their utilization in breeding programs. Over the years traditional breeding strategies have successfully incorporated novel alleles into elite germplasm, which has had significant impacts on production globally. A recent example is the development and release of biofortified wheat ‘Zinc Shakti (Chitra),’ developed by introgressing synthetic hexaploid (*A. tauschii* background) with elite germplasm, which has 40% higher grain Zn (Velu et al., 2015). Technologies such as GWAS and MABC are currently being used to explore the diversity and incorporate novel alleles into elite lines, though the lack of well-characterized genes and their closely linked markers impedes the process. GS and cisgenesis are promising technologies that could help harness large numbers of favorable exotic alleles and subsequently transfer them to elite backgrounds. Initiatives for genotyping and phenotyping of genetic resources through the gene banks are required to harness diversity efficiently and utilize in the breeding for improved wheat varieties.

## Author Contributions

RS: invited author; contributed to genetic diversity in grain yield and disease resistance, breeding approaches for harnessing diversity; SM: corresponding author; Tasks: compiling the review manuscript and contribution to following topics, genetic diversity for grain yield, climate resilience, traditional breeding approaches, high throughput phenotyping and cisgenesis; JR: harnessing genetic diversity through Genome wide association studies, marker assisted backcrossing, and genomic selection; GV: genetic diversity for Zinc and Iron and breeding approaches for Biofortified wheat; PS: genetic diversity for disease resistance (other than Wheat rust); XH: genetic diversity for disease resistance (other than Wheat rust); LC-H: genetic diversity for pest resistance, grain yield and breeding approaches; CG: genetic diversity for grain composition and end-use quality; SB: genetic diversity for rust resistance (Stem rust, yellow rust); CL: genetic diversity for rust resistance (Yellow rust, leaf rust).

## Conflict of Interest Statement

The authors declare that the research was conducted in the absence of any commercial or financial relationships that could be construed as a potential conflict of interest.

## Footnotes

1 www.rusttracker.org
